# Genome-Based Characterization of Emergent Invasive Neisseria meningitidis Serogroup Y Isolates in Sweden from 1995 to 2012

**DOI:** 10.1128/JCM.03524-14

**Published:** 2015-06-18

**Authors:** Bianca Törös, Sara T. Hedberg, Magnus Unemo, Susanne Jacobsson, Dorothea M. C. Hill, Per Olcén, Hans Fredlund, Holly B. Bratcher, Keith A. Jolley, Martin C. J. Maiden, Paula Mölling

**Affiliations:** a Department of Laboratory Medicine, Faculty of Medicine and Health, Örebro University, Örebro, Sweden; b Department of Zoology, University of Oxford, Oxford, United Kingdom

## Abstract

Invasive meningococcal disease (IMD) caused by Neisseria meningitidis serogroup Y has increased in Europe, especially in Scandinavia. In Sweden, serogroup Y is now the dominating serogroup, and in 2012, the serogroup Y disease incidence was 0.46/100,000 population. We previously showed that a strain type belonging to sequence type 23 was responsible for the increased prevalence of this serogroup in Sweden. The objective of this study was to investigate the serogroup Y emergence by whole-genome sequencing and compare the meningococcal population structure of Swedish invasive serogroup Y strains to those of other countries with different IMD incidence. Whole-genome sequencing was performed on invasive serogroup Y isolates from 1995 to 2012 in Sweden (*n* = 186). These isolates were compared to a collection of serogroup Y isolates from England, Wales, and Northern Ireland from 2010 to 2012 (*n* = 143), which had relatively low serogroup Y incidence, and two isolates obtained in 1999 in the United States, where serogroup Y remains one of the major causes of IMD. The meningococcal population structures were similar in the investigated regions; however, different strain types were prevalent in each geographic region. A number of genes known or hypothesized to have an impact on meningococcal virulence were shown to be associated with different strain types and subtypes. The reasons for the IMD increase are multifactorial and are influenced by increased virulence, host adaptive immunity, and transmission. Future genome-wide association studies are needed to reveal additional genes associated with serogroup Y meningococcal disease, and this work would benefit from a complete serogroup Y meningococcal reference genome.

## INTRODUCTION

Invasive meningococcal disease (IMD) remains, partly due to its rapid onset, lethal in about 10% of the cases in developed countries (1, 2). The annual incidence rates of IMD range from <1/100,000 population in low-incidence countries to >1,000/100,000 population during epidemics in sub-Saharan Africa (3, 4), and incidence is considered dependent on a combination of bacterial, host, and environmental factors. Meningococci expressing capsular polysaccharides corresponding to serogroups A, B, C, X, W, and Y are most commonly associated with IMD, although their global distribution varies (5). Serogroup Y meningococci are frequently carried but are considered to be less invasive than other serogroups, such as A, B, and C (5); however, serogroup Y meningococci belonging to multilocus sequence typing (MLST) sequence type 23 (ST-23) clonal complex (cc) increased in the United States during the mid-1990s (6) and caused around a third of all IMD cases in the United States until 2013 (http://www.cdc.gov/abcs/reports-findings/survreports/mening12.pdf). An increased incidence of serogroup Y IMD has also been noted in Europe from the late 2000s, mainly in Scandinavia (7, 8). Sweden had the highest incidence of serogroup Y IMD (0.46/100,000 population from an incidence of 1.1/100,000 for all IMD) and relative proportion (49%) of serogroup Y IMD in Europe in 2012 (9). In England and Wales, a slight increase in serogroup Y IMD has been noted (10), representing 10.2% of all IMD cases in 2012 (http://www.webarchive.org.uk/wayback/archive/20130329094507/http://www.hpa.org.uk/hpr/archives/2013/hpr0813.pdf). The average annual incidence of IMD is 2.0/100,000 in England and Wales (11).

Isolate characterization using MLST, and the genes coding for Porin A (*porA*), Ferric enterobactin transport protein A (*fetA*), Porin B (*porB*), Factor H binding protein (*fHbp*), and penicillin-binding protein 2 (*penA*), of all serogroup Y IMD isolates from 2000 to 2012 in Sweden indicated that a limited number of strain types was responsible for most IMD cases. Sixty-two percent of all serogroup Y isolates belonged to the three most prevalent strain types found in Sweden, referred to as clones YI through YIII, all of which were ST-23 (9). The most predominant strain type (YI) appeared to be responsible for the serogroup Y increase in Sweden (9, 12).

The development and application of highly parallel (next-generation) sequencing technology enables the analysis of large numbers of meningococci at the whole-genome sequencing (WGS) level. Here, the serogroup Y emergence and spread in Sweden was investigated using WGS data by the characterization of all invasive serogroup Y isolates from 1995 to 2012. This enabled the comparison of these isolates with serogroup Y genomes from elsewhere, including a large collection of serogroup Y isolates from England, Wales, and Northern Ireland, which has a relatively low serogroup Y IMD incidence, and two isolates from the United States, where serogroup Y remains one of the major causes of IMD.

## MATERIALS AND METHODS

### Bacterial isolates.

All invasive cases of meningococcal disease according to the European Union case definition (http://eur-lex.europa.eu/LexUriServ/LexUriServ.do?uri=OJ:L:2012:262:0001:0057:EN:PDF) are mandatorily reported by clinicians to the Public Health Agency of Sweden, and the corresponding isolates are sent to the Swedish Reference Laboratory for Pathogenic Neisseria. Capture-recapture analyses performed at our laboratory (data not published) have shown that the proportion of noncaptured cases is below 5%. The isolates were grown on chocolate agar at 37°C in a 5% CO_2_-enriched atmosphere overnight and archived at −70°C in preservation medium. The IMD isolates represented 97% of all serogroup Y cases reported in Sweden from January 1995 to November 2012. Basic epidemiological data (clinical site of isolation, age, gender, and area of residence) were available for all isolates. The collection comprised 186 out of the 188 (99%) serogroup Y culture-confirmed isolates identified during this period and had been isolated from blood (*n* = 150), cerebrospinal fluid (*n* = 32), and joint fluid (*n* = 4). All isolates were cultured and stored as part of routine diagnostics (standard care), and no patient identification information was used in the present study. Isolates from 2000 to 2012 had previously been characterized using MLST and PorA, FetA, *porB*, *fHbp*, and *penA* typing (9, 12).

Previously determined genome sequences were available from the Neisseria PubMLST website (http://pubmlst.org/neisseria/). Among these, serogroup Y isolates from the Meningitis Research Foundation Meningococcus Genome Library (MRF MGL), which included isolates from IMD surveillance in England, Wales, and Northern Ireland between epidemiological years 2010 to 2011 (*n* = 74) and 2011 to 2012 (*n* = 69), were used for comparison. This data set represents approximately 43% of all reported IMD cases in England, Wales, and Northern Ireland during the study period (13). In addition, the genome sequences of two predominant strain types in the early and late 1990s (NM220 and NM233, respectively) from the United States (isolated in Maryland in 1999) were included (6). The genetic profile of the early strain type (NM220) was Y:P1.5-1,2-2:F5-8:ST-23 (cc23) and of the late strain type (NM233) was Y:P1.5-2,10-1:F4-1:ST-1621 (cc23).

### Genome sequencing, assembly, and annotation.

For the Swedish isolates, genomic DNA (5 μg) was isolated from bacterial cultures using the Wizard genomic DNA purification kit (Promega) according to the manufacturer's instructions. Sequencing of purified DNA was performed by the Oxford Genomics Centre, Wellcome Trust Centre for Human Genetics, University of Oxford, United Kingdom. Samples were quantified using PicoGreen (Invitrogen), and the sample integrity was assessed using 1% E-Gel EX (Invitrogen). DNA was fragmented using an Episonic system with a process time of 200 s and 10 duty cycles. The distribution of fragments after shearing was determined using a Tapestation D1200 system (Agilent). The libraries were constructed using the NEBNext DNA sample prep master mix set 1 kit (New England BioLabs) with minor modifications. Ligation of the adapters was performed using Illumina adapters (multiplexing sample preparation oligonucleotide kit). The ligated libraries were size selected using AMPure magnetic beads (Agencourt) and were PCR enriched. Enrichment and adapter extension of each preparation were obtained using 5 μl of a size-selected library in a 50-μl PCR. After 10 cycles of amplification (cycling conditions per Illumina recommendations), the reactions were purified with AMPure XP beads (Agencourt Bioscience Corporation). The final size distribution was determined using a Tapestation 1DK system (Agilent), and the concentrations used to generate the multiplex pool were determined by PicoGreen (Invitrogen). The pooled libraries were quantified using the Agilent qPCR library quantification kit and an MX3005PTM instrument (Agilent). Sequencing was performed on the Illumina HiSeq 2000 sequencing system using paired-end 100-base reads with 20× to 50× coverage.

The short read sequences were *de novo* assembled using an automated assembly pipeline developed by James Bray (Oxford University, Department of Zoology), which utilizes the Velvet assembly package (14). The draft genomes were assembled and uploaded to the PubMLST database, which runs on the bacterial isolate genome sequence database (BIGSdb) platform (15). The genomes were automatically scanned against alleles defined in the sequence definition database using the BIGSdb software. New alleles were manually scanned, curated, and assigned new allele numbers.

### Genome comparisons.

The genomes were analyzed and compared using the BIGSdb genome comparator tool implemented within the PubMLST website (http://pubmlst.org/neisseria/) using the gene-by-gene analysis approach (16). Phylogenetic analysis of the isolates included 53 ribosomal protein genes (by ribosomal MLST [rMLST]) (17) and 1,600 loci defined as the core genome in the PubMLST Neisseria database (18). The genome comparator module automatically generated a list of loci and their corresponding allele numbers and information regarding which loci had sequence differences among isolates, were exactly matching among isolates, were incompletely assembled, or were missing in some isolates (19). Loci that were incomplete, due to the situation of the locus at the end of a contig, or those that were missing in at least one isolate were automatically removed from the distance matrix calculation for the Neighbor-Net graphs. A distance matrix was generated, based on the number of alleles differing between each isolate, and visualized in a Neighbor-Net graph (19) using SplitsTree4, available at http://www.splitstree.org/ (20). Support for sublineages was corroborated in Neighbor-Net graphs calculated from variable nucleotide sites of concatenated core genome locus alignments: The genome comparator produced MAFFT alignments of variable loci which were exported to MEGA6 (21) for extraction of variable sites, networks were created in SplitsTree4, and bootstrap analyses (1,000 replicates) were performed to provide support for the splits among sublineages.

For the comparison of virulence gene content, the list of 177 Neisseria genes known or hypothesized to have virulence properties, created by Marri et al. (22), were used.

### Nucleotide sequence accession numbers.

Isolate genomes are available at http://pubmlst.org/neisseria/ under the identification numbers 26064-66, 26068-81, and 26083-26251. Sequence reads have also been deposited in the European Nucleotide Archive (ENA), EMBL, under accession numbers ERR405852 to ERR405854, ERR405856 to ERR405869, and ERR405871 to ERR406038.

## RESULTS

### Quality of the genome assemblies.

Draft genomes were obtained from 185 of the 186 isolates (one isolate failed to sequence due to poor DNA quality). Once assembled, the draft genomes were not further manipulated. The number of contigs per genome assembly varied from 139 to 354, and the *N*_50_ values were between 17,194 and 62,933 base reads. The average *N*_50_, a value that represents the length at which contigs of equal or longer length contain at least 50% of the assembled sequence, provides an indication of the total genome coverage; however, it was not used as a measure of genome assembly quality. Overall, 359 loci in the Swedish genome collection and 411 loci of the MRF MGL and U.S. genomes were incomplete in some of the genomes and, therefore, were automatically removed from the distance matrix calculation for the Neighbor-Net graphs.

### Genome comparisons.

The genomes were compared to (i) the previous characterization using 12 genes (MLST, *fetA*, *fHbp*, *penA*, *porA*, and *porB* genes) of the 160 serogroup Y Swedish isolates from 2000 to 2012 (9, 12), (ii) the draft genomes of the 143 serogroup Y isolates included in the MRF MGL from England, Wales, and Northern Ireland during 2010 to 2011 and 2011 to 2012; and (iii) the genomes of the two serogroup Y strain types dominating in the United States during the 1990s (6).

The Swedish isolates belonged to ST-23 clonal complex, ST-167 clonal complex, and ST-174 clonal complex (four genomes were unassigned); 175 of 185 (95%) of the isolates belonged to the ST-23 clonal complex. There were, in total, 35 fine types (identical PorA VR1, VR2, FetA VR, and MLST ST); 51% of the isolates belonged to fine type Y:P1.5-2,10-1:F4-1:ST-23 (cc23).

Different serogroup Y strain types (identified using the 12 typing loci) (9, 12) predominated in Sweden, in England, Wales, and Northern Ireland, and in the United States among the genomes investigated (Table 1). However, all of them belonged to the ST-23 clonal complex. Only 12 isolates from the MRF MGL, and strain type NM220 from the United States (6), had typing profiles that matched the predominant Swedish strain types (Table 1). The two predominant strains in England, Northern Ireland, and Wales; Y:P1.5-1,10-1:F4-1:ST-1655 (cc23) and Y:P1.5-1,10-4:F4-1:ST-1655 (cc23) were rare in the Swedish serogroup Y population (*n* = 2). A ribosomal MLST (rMLST) (17) Neighbor-Net graph of the Swedish genomes clustered the previously described strain types YI, YII, and YIII (9, 12) separately from each other and showed a similar topology to an rMLST Neighbor-Net graph of the genomes from England, Wales, and Northern Ireland and the United States (data not shown).

**TABLE 1:** Genetic profiles for the most prevalent serogroup Y strain types in Sweden (1995–2012), their respective frequency among isolates in England, Wales, and Northern Ireland (2010–2011 and 2011–2012), and the two strain types from the United States during the 1990s^*a*^

Strain type	Fine typing^*b*^			Extended fine typing				Whole-genome lineage	Isolate frequency (%)		
	PorA VR1, VR2	FetA VR	ST (cc^*c*^)	′*porB^*d*^*	PorA VR3	*fHbp*	′*penA*^*d*^		Sweden	England, Wales, NI^*e*^	USA
YI	P1.5-2,10-1	F4-1	ST-23 (cc23)	3-36	36-2	25	22	23.1	83/185 (45)	3/143 (2.1)	0
YII	P1.5-1,2-2	F5-8	ST-23 (cc23)	2-55	36-2	25	22	23.2	18/185 (9.7)	3/143 (2.1)	1/2 (0.5)
YIII	P1.5-1,2-2	F5-8	ST-23 (cc23)	3-36	36-2	25	1	23.2	7/185 (3.8)	6/143 (4.2)	0
YIV	P1.5-2,10-1	F5-12	ST-23 (cc23)	3-36	36-2	25	22	23.1	6/185 (3.2)	0	0

a U.S. strain types described in reference 6.

b VR, variable region; ST, sequence type.

c CC, clonal complex.

d Partial fragment.

e Northern Ireland.

**FIG 1:**
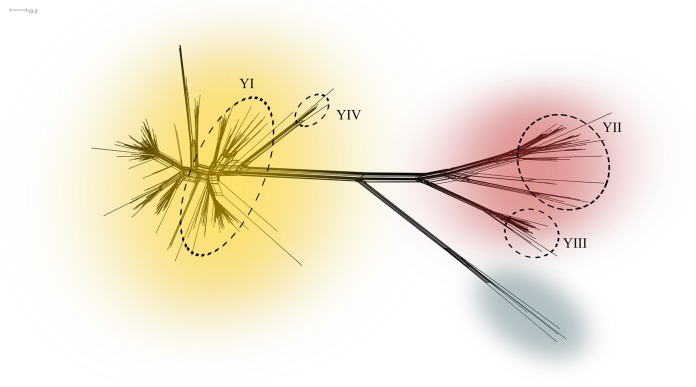
Neighbor-Net graph of all Neisseria meningitidis ST-23 clonal complex core genomes in the PubMLST database (*n* = 434) constructed with 767 of 1,600 core genes. Sublineages 23.1, 23.2, and 23.3 are shown in yellow, rust, and gray, respectively. The most common invasive N. meningitidis serogroup Y strain types (characterized by 12 genes) in Sweden, YI, YII, YIII (9, 12), and YIV, are found among the isolates within the dashed circles.

Core genome MLST (cgMLST) analysis of all 767 of the 1,600 loci that were complete in all genome assemblies present in the PubMLST database belonging to the ST-23 clonal complex (lineage 23) at the time of writing (*n* = 434; 376 serogroup Y isolates, 1 serogroup W isolate, and 57 isolates that were unassigned or nongroupable) visually revealed three well-supported sublineages, 23.1 (*n* = 300), 23.2 (*n* = 60), and 23.3 (*n* = 4) (Fig. 1; see Fig. S1 in the supplemental material). Isolates from Sweden, England, Wales, and Northern Ireland represented 97% of all the ST-23 clonal complex isolates in PubMLST, and there were approximately 500 to 600 loci with alleles that differed between sublineages 23.1 and 23.2. Swedish genomes with the previously defined strain types YI, YII, and YIII belonged to sublineages 23.1, 23.2, and 23.2, respectively.

**FIG 2:**
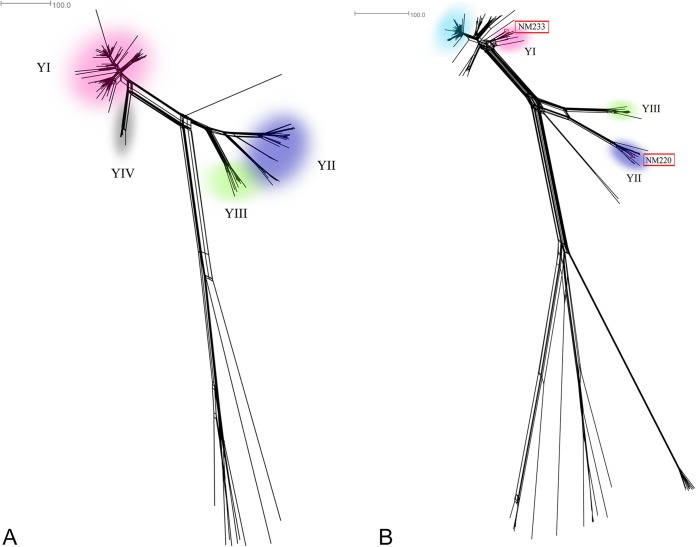
Neighbor-Net graphs constructed with 1,241 of 1,600 and 1,189 of 1,600 loci defined as the core genome in the PubMLST database (A and B, respectively). The Neisseria meningitidis serogroup Y isolates were collected in (A) Sweden, 1995 to 2012 (*n* = 185), and (B) England, Wales, and Northern Ireland, 2010 to 2011 and 2011 to 2012 (*n* = 143), and Maryland, 1999 (strain types NM220 and NM233 [6], marked in red boxes). Isolates belonging to the most common invasive N. meningitidis serogroup Y strain types (characterized by 12 genes) in Sweden, YI, YII, YIII (9, 12), and YIV, are found among the isolates in the pink, purple, green, and gray areas, respectively. Isolates belonging to the predominating strain type in England, Wales, and Northern Ireland (Table 1) are found among the isolates in the light blue area. The genetic distance was defined as number of loci with allelic differences.

The Swedish serogroup Y meningococcal population was further resolved by cgMLST analysis, revealing a fourth possible strain type (Fig. 2A). This fourth strain type, YIV, comprised six isolates collected from 2004 to 2010, mainly in the western parts of Sweden, and had the same MLST, PorA, ′*porB* (truncated), *fHbp*, and *′penA* types, but a different FetA F5-12, as strain type YI (Table 1). It was a member of sublineage 23.1 (Fig. 1). Moreover, isolates belonging to the strain YII were differentiated into two clusters by cgMLST (Fig. 2A). These clusters were separated by approximately 300 loci with allele differences and by the years of collection: One cluster included only isolates from 1995 to 2002 and the other, only isolates from 2007 to 2012. Strain types YII and YIII were separated by approximately 380 loci with allele differences, and isolates belonging to other clonal complexes were clearly separated by allelic differences in more than 900 loci.

**FIG 3:**
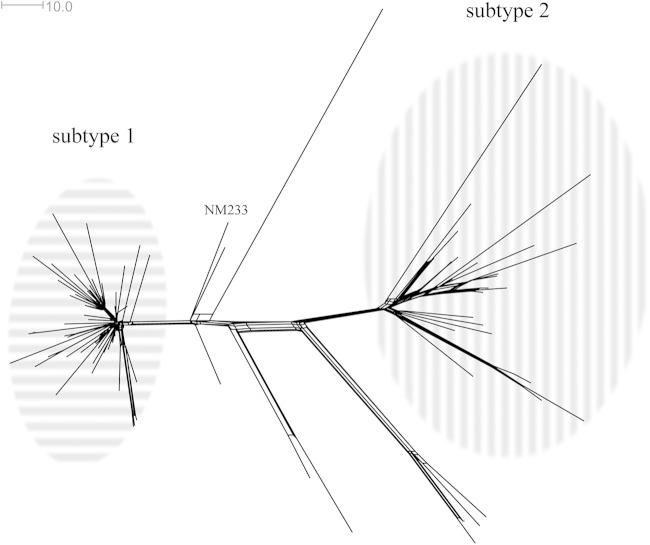
Neighbor-Net graph constructed with 1,387 of 1,600 core genes from all Swedish Neisseria meningitidis strains belonging to the predominant serogroup Y strain type YI (9, 12) collected from 1995 to 2012 (*n* = 83). The two main clusters (assessed visually) are referred to as subtype 1 (*n* = 52) and subtype 2 (*n* = 23) and have been circled in horizontal and vertical stripes, respectively. Three isolates from the United Kingdom which share the same genetic profile as the Swedish predominant serogroup Y strain type YI have also been included. The serogroup Y strain NM233 from Maryland is representative of the dominating strain type from the late 1990s in the United States (6). The genetic distance was defined as the number of loci with allelic differences.

**FIG 4:**
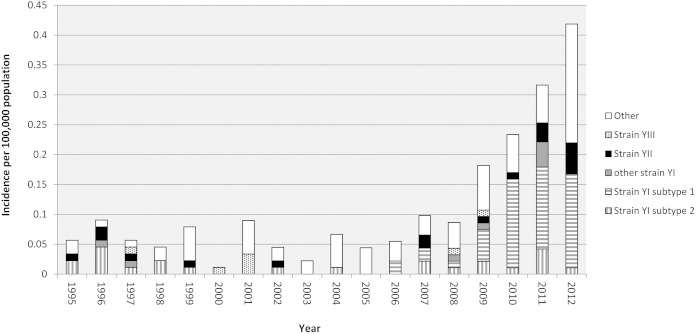
Distribution of the two subtypes of the predominant serogroup Y strain type YI, all other YI isolates, the second-most-common (YII) and third-most-common (YIII) strain types (9, 12), and all other serogroup Y isolates in Sweden, 1995 to 2012.

The core genome analysis of only Swedish strain type YI isolates (Fig. 3) revealed two clusters, separated by approximately 100 loci with allele differences. They visually appeared to be subtypes of the predominant strain type YI, and were referred to as subtypes 1 and 2 (Fig. 3). The isolates in the different subtypes were also separated by the years of collection: Subtype 2 contained isolates from all years investigated (1995 to 2012), while subtype 1 was limited to isolates collected after 2006 (Fig. 4). There was no statistically significant difference (*P* > 0.05, Mann-Whitney U test) in the age of the patients with IMD or in geographical differences between the different subtypes (data not shown). Only one of the three isolates from the United Kingdom with a strain type YI profile (collected in the northwest area of the country in 2012) clustered with any of the isolates in subtypes 1 and 2, namely, the ones in subtype 1. Furthermore, strain type NM233 from the United States (6), included in the same Neighbor-Net graph as the Swedish YI isolates, (Fig. 3), did not cluster with any of the subtypes, although it was a member of sublineage 23.1 (Fig. 1). Approximately 70 and 120 loci with allele differences were identified between NM233 and subvariant 1 and 2, respectively. Only 1,387 of the 1,600 loci in the core genome were included in the analysis, as the remaining 213 loci were incompletely assembled.

In 2012, the diversity among serogroup Y isolates in Sweden had clearly increased compared to recent years, and isolates that did not belong to the most frequent strain types (YI through YIII) represented 47.5% (19 of 40 isolates) of all isolates that year (Fig. 4). These isolates were highly diverse, with only 3 of 19 isolates sharing the same genetic typing profile (using the 12 genes previously described); each of the remaining 16 had unique profiles.

### Genomic variation.

The gene *nadA* (locus NEIS1969) failed to assemble in all genomes belonging to lineage 23 (ST-23 clonal complex) but contained the 16-nucleotide sequence 5′-TTTCCATTCCAAACGC-3′, which is common in *nadA*-deficient strains (23). Two loci, NEIS1094 (*cysD*) and NEIS1342, in the sulfate adenylyltransferase subunit 2 and cytolysin secretion ABC transporter, respectively, failed to assemble in all strain type YI genomes but were assembled in all YII and YIII genomes. Similarly, one locus, NEIS0627 (coding for a hypothetical protein) could not be identified in any of the genomes belonging to strain YI subtype 1 but was found in all genomes belonging to subtype 2.

The hypothesized virulence genes in Neisseria (22, 24–26), among all genes in the Neisseria PubMLST website (http://pubmlst.org/neisseria/) differing between lineage 23.1 and 23.2, are shown in Table 2. Potential virulence genes differing between the two subtypes of strain type YI in the Swedish isolates are also shown in Table 3. Internal stop codons were found in the *lpxL* (NEIS1351) gene of Swedish strain types YI and YII (data not shown) and in the locus NEIS1965 (coding for a putative inner membrane transport protein) of Swedish subtype 1 and the gene *opcA* (NEIS2198) of Swedish subtype 2 (Table 3).

**TABLE 2:** Loci with allelic differences between sublineages 23.1 (*n* = 300) and 23.2 (*n* = 60) among genes hypothesized to play a role in meningococcal virulence^*a*^

BIGSdb Neisseria locus identifier	Predicted protein/function (gene)	Allele no. (% of isolates with this allele):		% nucleotide identity	No. of amino acid differences
		23.1	23.2		
NEIS0537	Two-component system regulator; *ompR* family	2 (100)	1 (100)	99.6	0
NEIS1853	Multidrug resistance translocase (*farA*)	148 (97)	10 (98)	95.1	35
NEIS1852	Multidrug resistance translocase (*farB*)	117 (92)	130 (98)	98.1	4
NEIS0577	Iron-uptake permease inner membrane protein (*fbpB*)	1 (98)	5 (98)	99.8	0
NEIS1963	Enterobactin receptor (*fetA*)	223 (95)	30 (82)	49.1	42
NEIS0624	3-Deoxy-manno-octulosonate cytidylyltransferase (*kdsB*)	33 (99)	14 (100)	99.9	1
NEIS0899	Alpha-2,3-sialyltransferase/LOS^*b*^ alpha chain transferases (*lst*)	241 (90)	25 (95)	99.91	1
NEIS0528	Periplasmic binding protein	3 (99)	38 (93)	98	4
NEIS0661	Hypothetical protein	29 (100)	2 (100)	99.8	0
NEIS0612	Outer membrane protein (*nspA*)	92 (99)	17 (100)	99.8	1
NEIS1632	Outer membrane lipoprotein (*mtrE*)	62 (96)	5 (98)	97.9	4
NEIS0487	Type IV pilin/pilus-associated protein	102 (89)	48 (80)	88.7	22

a From the collection of 177 putative Neisseria virulence genes described in reference 22.

b LOS, lipooligosaccharide.

**TABLE 3:** Loci with allelic differences of genes hypothesized to play a role in meningococcal virulence^*a*^ between the two subtypes of the predominant strain type YI

BIGSdb Neisseria locus identifier	Predicted protein/function (gene)	Allele no. (% of isolates with this allele)		% Nucleotide identity	No. of amino acid differences
		Subtype 1	Subtype 2		
NEIS0048	UDP-glucose epimerase/LPS^*b*^ synthesis (*galE*)	38 (100)	169 (100)	99.1	0
NEIS0669	Ferrochelatase (*hemH*)	45 (100)	9 (100)	95.4	13
NEIS1902	Lacto-*N*-neotetraose biosynthesis glycosyl transferase/LOS^*c*^ alpha chain transferases (*lgtA*)	79 (98)	22 (100)	99.0	2
NEIS1901	Lacto-*N*-neotetraose biosynthesis glycosyl transferase/LOS alpha chain transferases (*lgtB*)	81 (98)	24 (100)	96.4	13
NEIS0421	Lipid A biosynthesis lauroyl transferase (*lpxL2*)	70 (100)	11 (100)	99.9	1
NEIS1513	AraC family transcriptional regulator (*mtrA*)	43 (98)	6 (100)	99.9	0
NEIS1634	Membrane fusion protein (*mtrC*)	88 (96)	41 (100)	99.9	1
NEIS1965	Putative inner membrane transport protein	279^*d*^ (100)	61 (100)	99.9	Frameshift
NEIS2198	Outer membrane adhesin (*opcA*)	18 (94)	86^*d*^ (100)	99.9	Frameshift
NEIS0396	Pilin glycosylation protein (*pglD*)	51 (100)	200 (83)	100.0	1

a From the collection of 177 putative Neisseria virulence genes described in reference 22.

b LPS, lipopolysaccharide.

c LOS, lipooligosaccharide.

d Allele sequence contained an internal stop codon.

## DISCUSSION

For reasons that remain incompletely understood, the incidence of invasive meningococcal disease is highly variable around the world, ranging from low levels of endemicity in most areas at most times through localized hyperendemicity and epidemic outbreaks and to national, regional, and occasionally pandemic disease. It is thought that these outbreaks are due to an interaction of distinct meningococcal lineages, the hyperinvasive lineages, with human host populations although the details of these interactions are yet to be resolved. Each of these lineages has a characteristic epidemiological behavior and is associated with, and identified by, particular antigen repertoires. Here, we have used whole-genome sequence (WGS) data to investigate the prolonged increase in the incidence of serogroup Y disease in Sweden caused by meningococci belonging to the ST-23 clonal complex. WGS data from multiple isolates enabled a high-resolution examination of the phylogenetic and antigenic relationships for the Swedish isolates to related meningococci isolated in other countries.

In the 1990s and late 2000s, serogroup Y meningococci increased in incidence in North America and Europe, respectively, with a particularly marked increase in Sweden. In the period before this, serogroup Y meningococcal disease was relatively rare and particularly associated with disease in older individuals. Analyses with MLST and antigen sequencing demonstrated that most serogroup Y isolates from this increase in disease belonged to the ST-23 clonal complex, which has a relatively low invasive capacity compared to other hyperinvasive lineages. These studies demonstrated that there was a succession of two antigenically distinct strains in North America (late and early strains) and at least three related but distinct strains causing disease in Sweden (referred to as YI through YIII) (9, 12). The serogroup Y disease increase was not, therefore, attributable to the spread of a single virulent variant but was caused by a number of related cocirculating members of the ST-23 clonal complex, similar to those seen elsewhere and which were widely distributed throughout Sweden over a prolonged period.

Analysis of the WGS data Swedish isolates at the rMLST (data not shown) and cgMLST loci demonstrated two major branches in the phylogeny of the Swedish isolates belonging to the ST-23 clonal complex (Fig. 2), one of which largely corresponded to the YI strain, with another containing distinct clusters corresponding to the YII and YIII strain types (Fig. 2A). Comparison with other isolates showed that the Swedish isolates reflected the diversity seen in the United Kingdom and elsewhere (Fig. 2B), as recorded in the PubMLST database (http://pubmlst.org/neisseria/). The first cluster (lineage 23.1) which contained the Swedish YI isolates also included the genome-sequenced U.S. isolate NM233, which is representative of the late U.S. serogroup Y strains. The second major cluster (lineage 23.2) included the Swedish YII and YIII strain types and the genome-sequenced U.S. isolate NM220, representative of the early U.S. serogroup Y isolates. This confirmed that both of these types have a distribution spanning North America and Europe, including Sweden, over a number of years.

The automated extraction of the antigen gene and other typing loci demonstrated that, while many of the members of a particular cluster shared the same strain type, each cluster contained numerous variants (see Table S1 in the supplemental material). Most of these variants differed at one or, at most, a few loci, and the majority occurred only once in the Swedish isolate collection (see Table S1). An exception was a cluster closely related to that corresponding to the YI strain, tentatively called YIV, which contained 8 isolates isolated over a period of 7 years, sharing 1,684 identical loci and differentiated by 137 variable loci in a cgMLST analysis. In conclusion, while conventional typing can identify lineages and variants within them with good discrimination, WGS data are required to definitively establish precise relationships among closely related members of this highly diverse bacterium, which has implications for epidemiological and outbreak analysis. It is particularly important that minor variants obtained by WGS, which are derived from major clones, are not excluded from epidemiological analyses of major strain types (see Table S1).

Most of the increase in serogroup Y meningococcal disease in Sweden was caused by strain type YI (belonging to WGS lineage 23.1). A cgMLST analysis of these isolates showed them to separate into two distinct clusters, which were antigenically indistinguishable but distinct at the cgMLST analysis (subtypes 1 and 2)(Fig. 3). Interestingly, the temporal distribution of these two clusters in Sweden coincided with the start of the increase in serogroup Y meningococcal infections around 2006 (Fig. 4); strain YI subtype 1 appeared in the Swedish isolate collection only after 2006, and these isolates were largely responsible for the increase in serogroup Y meningococcal disease. These closely related isolates were similar to, but distinct from, the U.S. isolate NM233, representative of the late strain observed in the United States, which was responsible for an increase in the incidence in the United States and which was distributed through Sweden. Thus, while the serogroup Y ST-23 disease in Sweden was highly diverse and similar to that seen in other countries, there is some evidence that the increase in disease from 2009 to 2011 was largely due to a single subtype and may reflect the introduction and spread of this variant within the population. However, in contrast to the epidemiology observed in the United States, this subtype was not different from the related strain YI subtype 2 in its major antigens. Consequently, antigenic shift, at least in the typing antigens, could not be invoked as an explanation for the increase in this clone; therefore, if this increase was indeed due to differences between these clusters, these will have to be sought elsewhere in the genome.

A collection of 177 putative Neisseria virulence genes (22) were used to investigate potential differences in virulence among the Swedish serogoup Y isolates. Of these, 24 genes could not be included, as they were paralogous to other genes in the genome. Consistent with previous studies on serogroup Y isolates (6, 10), *nadA* (NEIS1969) was absent in all the ST-23 clonal complex genomes studied here. This adhesin has been shown to be present in at least 50% of disease-associated strains and in 98% of all isolates belonging to four hypervirulent serogroup B and C strains (23). The genes *lpxL* (NEIS1351), NEIS1965, and *opcA* (NEIS2198), considered to play a role in meningococcal virulence (22), were present as pseudogenes in some of the strain types or subtypes (Tables 2 and 3). All lineage strain type YI and YII isolates had the previously described (27) mutation V in the *lpxL* gene, and the strain type YII isolates had also mutation IV. These mutations in *lpxL* have been described to result in a low-activity lipopolysaccharide (LPS) which gives a defective Toll-like receptor 4 (TLR4) activation and evasion of the innate immune system (27). In addition, *opcA* is known to mediate the adhesion of meningococci to epithelial and endothelial cells by binding to cell-surface receptors (28, 29). Possibly, the presence of an *opcA* pseudogene in strain YI subtype 2 could result in lower adhesion and, thereby, less invasive capacity. The three genes which failed to assemble in all strain type YI or subtype 1 isolates, NEIS1094 (*cysD*), NEIS1342, and NEIS0627, have not previously been considered to contribute to the virulence of Neisseria species (22, 24–26). Many additional differences were found between the different strain types and subtypes, but these genes had not previously been associated with virulence and were not further considered. As not all of the genes contained in the Neisseria pathogenome have been defined and characterized, it is possible that further genes with an important role in the pathogenicity are yet to be described. Without a high-quality assembled genome, effects on the virulence of genes outside the core genome or among the excluded genes, which were incompletely assembled, cannot be excluded. Furthermore, alterations in the timing and level of gene transcription and translation and the effect of small-scale gene variation, such as amino acid alterations, could not be judged based on the present genomic and phenotypic data.

In conclusion, the population structure of the Swedish serogroup Y isolates was comparable to the isolate population structure from England, Wales, and Northern Ireland. There were also close similarities with isolates obtained in the United States and elsewhere, indicating that the serogroup Y ST-23 likely has a global distribution. However, there is also some evidence for differences in the incidence of particular variants at particular times. For example, a study of IMD isolates in the United States from 2000 to 2005 (30) indicated that the most common clone belonged to serogroup Y and shared the same fine type as the Swedish strain type YI, although it is difficult to assess if this clone is identical to YI in the absence of WGS data. We have shown that the expansion of serogroup Y disease in Sweden was mostly due to the increase of subtype 1 of the previously described strain type YI (9, 12). This expansion was, unlike the experience in the United States, not associated with an antigenic shift. Future genome-wide association studies, after establishing a reference pangenome for serogroup Y, are therefore necessary to reveal additional genes associated with serogroup Y meningococcal disease and in particular the Swedish strains.

## Supplementary Material

Supplemental material
