# Supplementary material for: Genome-Based Characterization of Emergent Invasive Neisseria meningitidis Serogroup Y Isolates in Sweden from 1995 to 2012
Source: J Clin Microbiol. 2015 Jun 18;53(7):2154–62. doi: 10.1128/JCM.03524-14 (PMC4473204; doi:10.1128/JCM.03524-14)
Supplement: Supplemental material [file supp_53_7_2154__index.html]

Supplemental material 

# Genome-Based Characterization of Emergent Invasive Neisseria meningitidis Serogroup Y Isolates in Sweden from 1995 to 2012

## Supplemental material

- Supplemental file 1 -

  Fig. S1 (Lineage 23 sublineages identified in neighbor-net graphs)

  PDF, 180K
- Supplemental file 2 -

  Table S1 (Fine-typing information on all Swedish *Neisseria meningitidis* serogroup Y isolates belonging to the ST-23 clonal complex, divided into different strain types)

  PDF, 445K
