# Supplementary material for: Genome-Based Characterization of Emergent Invasive Neisseria meningitidis Serogroup Y Isolates in Sweden from 1995 to 2012
Source: J Clin Microbiol. 2015 Jun 18;53(7):2154–62. doi: 10.1128/JCM.03524-14 (PMC4473204; doi:10.1128/JCM.03524-14)
Supplement: Supplemental material [file JCM.03524-14_zjm999094338so1.pdf]

A)

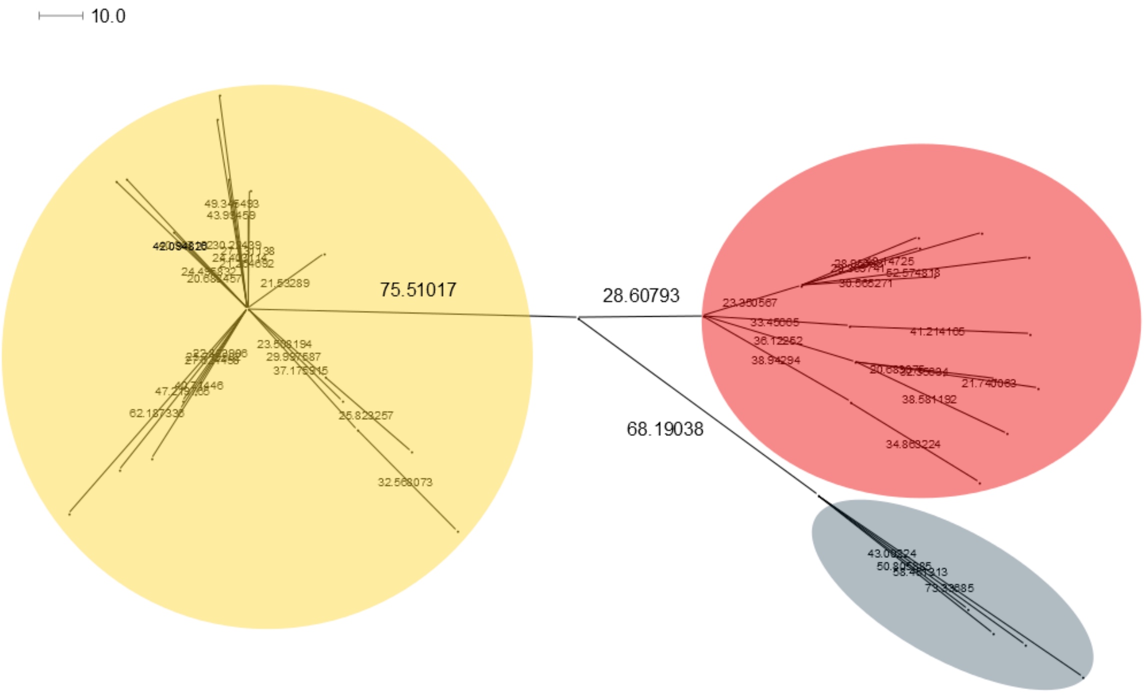

B)

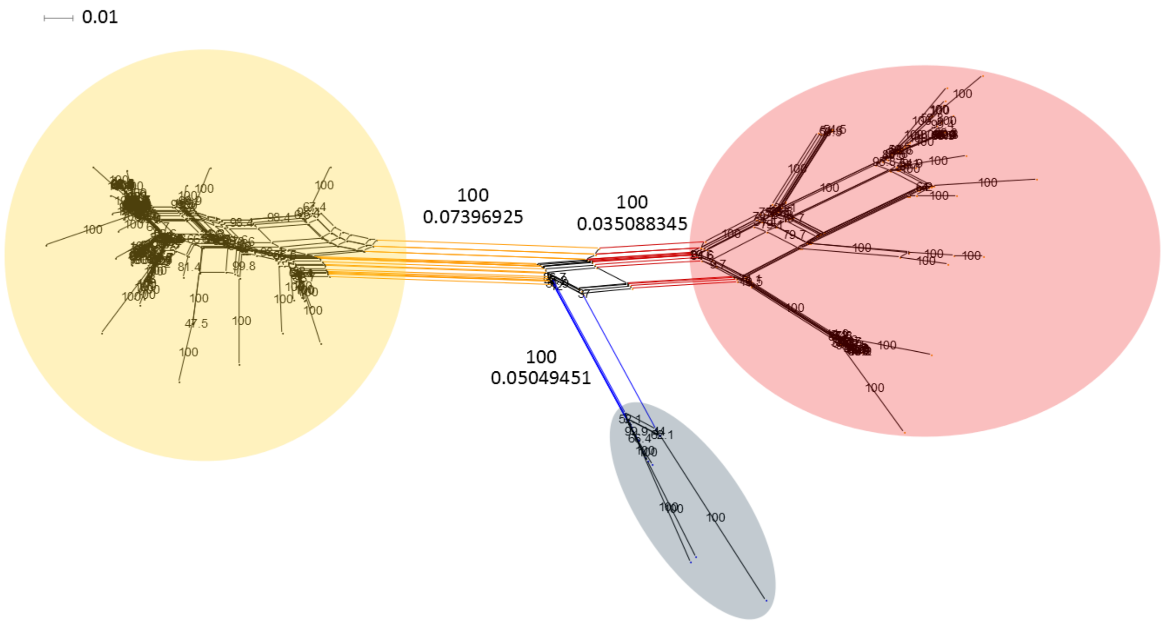

SupplementaryFigure 1. Lineage 23 sub-lineages identified in: A) Neighbor-Net graph created from allelic distances in 767 core genes among all *Neisseria meningitidis* ST-23 clonal complex genomes in the PubMLST database (n=434), showing only splits of weight >20 (weights annotated); and B) Neighbor-Net graph created

from variable nucleotide sites in the 767 genes (n=19,686) among a subset of all *N. meningitidis* ST-23 clonal complex core genomes in the PubMLST database (n=118). Bootstrap values annotated, with weights below for edges splitting sub-lineages. Sub-lineages 23.1, 23.2 and 23.3 are shown in yellow, rust and grey, respectively.
