# Supplementary material for: Genome-Based Characterization of Emergent Invasive Neisseria meningitidis Serogroup Y Isolates in Sweden from 1995 to 2012
Source: J Clin Microbiol. 2015 Jun 18;53(7):2154–62. doi: 10.1128/JCM.03524-14 (PMC4473204; doi:10.1128/JCM.03524-14)
Supplement: Supplemental material [file JCM.03524-14_zjm999094338so2.pdf]

Supplementary Table 1. Finetyping information on all Swedish *Neisseria meningitidis* serogroup Y isolates belonging to the ST-23 clonal complex divided into different strain types.

Strain type[a]	Frequency (%)	Finetyping				Extended finetyping				Whole-genome lineage
		ST[b] (MLST[c])	PorA VR[d]1	PorA VR[d]2	FetAVR[d]	PorA VR[d]3	*'porB*	*fHbp*	*penA*	
YI	80 (42.78)	23	5-2	10-1	F4-1	36-2	3-36	25	22	23.1
N/A	5 (2.67)	23	5-2	10-1	F4-1	36-2	3-36	25	*absent*	23.1
N/A	5 (2.67)	2692	5-2	10-1	F4-1	36-2	3-36	25	22	23.1
N/A	4 (2.14)	23	5-2	10-28	F4-1	36-2	3-36	25	22	23.1
N/A	3 (1.6)	23	5-2	10-1	F4-1	36-2	3-117	25	22	23.1
N/A	2 (1.07)	23	5-2	10-85	F4-1	36-2	3-36	25	22	23.1
N/A	2 (1.07)	23	5-2	10-12	F4-1	36-2	3-36	25	22	23.1
N/A	1 (0.53)	23	5-2	10-1	F4-1	36-2	3-36	25	14	23.1
N/A	1 (0.53)	23	5-2	10-1	F4-1	36-2	3-36	25	352	23.1
N/A	1 (0.53)	23	5-2	10-1	F4-1	36-2	3-36	276	22	23.1
N/A	1 (0.53)	23	5-2	10-1	F4-1	36-2	3-36	478	22	23.1
N/A	1 (0.53)	23	5-2	10-1	F4-1	36-2	3-454	25	22	23.1
N/A	1 (0.53)	23	5-2	10-1	F4-1	36-2	3-117	25	9	23.1
N/A	1 (0.53)	23	5-2	10-1	F4-1	36-2	3-36	144	*absent*	23.1
N/A	1 (0.53)	23	5-2	10-1	F3-22	36-2	3-36	25	*absent*	23.1
N/A	1 (0.53)	23	5-2	10-29	F4-1	36-2	3-36	25	22	23.1
N/A	1 (0.53)	23	5-2	10-12	F4-1	36-2	3-36	25	*absent*	23.1
N/A	1 (0.53)	23	5-2	10-51	F4-1	36-2	3-158	25	22	23.1
N/A	1 (0.53)	10213	5-2	10-28	F4-1	36-2	3-36	25	22	23.1
N/A	1 (0.53)	10098	5-2	10-1	F4-1	36-2	3-455	25	22	23.1
N/A	1 (0.53)	2692	5-2	10-3	F4-1	36-2	3-36	25	22	23.1
N/A	1 (0.53)	23	5	2	F4-1	36-2	3-36	25	22	23.1
YIV	6 (3.21)	23	5-2	10-1	F5-12	36-2	3-36	25	22	23.1
N/A	1 (0.53)	9299	5-2	10-1	F5-12	36-2	3-307	25	22	23.1
N/A	1 (0.53)	23	5-1	10-4[d]	F4-1	36-2	3-36	25	22	23.1
N/A	1 (0.53)	1655	5-1	10-4[d]	F4-1	36-2	3-36	25	22	23.1
N/A	1 (0.53)	1655	5-1	10-1	F4-1	36-2	3-117	25	22	23.1
YII	18	23	5-1	2-2	F5-8	36-2	2-55	25	22	23.2
N/A	5 (2.67)	23	5-1	2-2	F1-18	36-2	2-55	25	22	23.2
N/A	5 (2.67)	1625	5-1	2-2	F5-8	36-2	2-55	25	22	23.2
N/A	4 (2.14)	3801	5-1	2-2	F5-8	36-2	2-55	25	22	23.2
N/A	2 (1.07)	8043	5-1	2-2	F5-8	36-2	2-55	25	22	23.2
N/A	1 (0.53)	4988	5-1	2-2	-	36-2	3-36	25	22	23.2
N/A	1 (0.53)	23	5-1	2-2	F1-68	36-2	2-55	25	22	23.2
N/A	1 (0.53)	23	5-1	2-2	F5-8	36-2	2-55	25	342	23.2
N/A	1 (0.53)	10210	5-1	2-2	F1-18	36-2	2-55	25	22	23.2

**YIII**	8 (4.28)	23	5-1	2-2	F5-8	36-2	3-36	25	1	23.2
**N/A**	1 (0.53)	23	5-1	2-2	F5-8	36-2	3-117	25	1	23.2
**N/A**	1 (0.53)	23	5-1	2-2	F5-8	36-2	3-36	814	1	23.2
**N/A**	1 (0.53)	183	5-2	10-2	F4-1	36-2	3-53	104	22	23.3

N/A=not applicable

[a]Strain types are defined by isolates with identical sequence types, clonal complex, PorA variable regions 1,2 and 3, FetA variable region in combination with identical *'porB*, *fHbp* and *penA* alleles.

[b]sequence type

[c] clonal complex

[d]variable region
